# Analysis of the Urine Volatilome of COVID-19 Patients and the Possible Metabolic Alterations Produced by the Disease

**DOI:** 10.3390/metabo14110638

**Published:** 2024-11-19

**Authors:** Jennifer Narro-Serrano, Maruan Shalabi-Benavent, José María Álamo-Marzo, Álvaro Maximiliam Seijo-García, Frutos Carlos Marhuenda-Egea

**Affiliations:** 1 Department of Physical Chemistry, University of Alicante, 03690 Alicante, Spain; jennifer.narro@ua.es; 2 Hospital Marina Baixa, 03570 Alicante, Spain; shalabi_mar@gva.es (M.S.-B.); alamo_jos@gva.es (J.M.Á.-M.); 3 Department of Biochemistry and Molecular Biology and Soil Science and Agricultural Chemistry, University of Alicante, 03690 Alicante, Spain; amsg16@gcloud.ua.es

**Keywords:** COVID-19, SARS-CoV-2, urine volatilome, VOCs, GC-MS, metabolomics, biomarkers

## Abstract

Alterations in metabolism caused by SARS-CoV-2 infection have been highlighted in various investigations and have been used to search for biomarkers in different biological matrices. However, the selected biomarkers vary greatly across studies. Our objective is to provide a robust selection of biomarkers, including results from different sample treatments in the analysis of volatile organic compounds (VOCs) present in urine samples from patients with COVID-19. Between September 2021 and May 2022, urine samples were collected from 35 hospitalized COVID-19 patients and 32 healthy controls. The samples were analyzed by headspace (HS) solid phase microextraction (SPME) coupled to gas chromatography–mass spectrometry (GC-MS). Analyses were conducted on untreated urine samples and on samples that underwent specific pretreatments: lyophilization and treatment with sulfuric acid. Partial Least Squares Linear Discriminant Analysis (PLS-LDA) and Subwindow Permutation Analysis (SPA) models were established to distinguish patterns between COVID-19 patients and healthy controls. The results identify compounds that are present in different proportions in urine samples from COVID-19 patients compared to those from healthy individuals. Analysis of urine samples using HS-SPME-GC-MS reveals differences between COVID-19 patients and healthy individuals. These differences are more pronounced when methods that enhance VOC formation are used. However, these pretreatments can cause reactions between sample components, creating additional products or removing compounds, so biomarker selection could be altered. Therefore, using a combination of methods may be more informative when evaluating metabolic alterations caused by viral infections and would allow for a better selection of biomarkers.

## 1. Introduction

The COVID-19 pandemic emerged in 2019 and, since then, more than 775 million cases have been confirmed worldwide, according to the World Health Organization [1]. It has been observed that around a third of patients may present some type of pathology two years after passing the acute phase of the disease [2]. Therefore, although the state of emergency was suspended on 5 May 2023, the disease continues to affect a large portion of the population. The set of symptoms that remain after the disease has been referred to as “long-COVID” or “post-COVID-19” [3]. Several of these symptoms are directly related to metabolism, but the underlying mechanisms have not yet been elucidated. 

Among the strategies used to study metabolism, metabolomic studies stand out due to the large amount of information they provide. These studies can be conducted on different biological matrices, such as urine, blood, feces or breath. Urine is particularly useful because it is an easily accessible biofluid and has the advantage of being a concentrated biological sample.

Metabolomic studies have already been used in the analysis of COVID-19 patients using nuclear magnetic resonance (NMR) [4,5,6,7,8], high-performance liquid chromatography coupled with mass spectrometry (HPLC-MS) [9,10,11,12], or gas chromatography coupled with mass spectrometry (GC-MS) [13,14,15,16,17,18]. 

Various studies aim to distinguish COVID-19 patients from healthy individuals based on the analysis of volatile organic compounds (VOCs) [13,15,16,19,20,21,22,23,24]. However, the selected biomarkers vary greatly across investigations. To reduce errors in the subsequent chemometric analysis of VOCs, different techniques are often used to increase the signal of the peaks of interest and reduce interference from particles released by the fiber used in the GC-MS equipment. These techniques include adding a strong acid to promote protonation and the volatility of the compounds or lyophilizing the sample before analysis [25,26]. 

Our objective is to provide a more robust selection of biomarkers in the analysis of VOCs present in urine samples from COVID-19 patients, including results from different sample pretreatments. This is not a comparison of pretreatment methods but rather an integrated analysis of the results. To this end, VOCs present in urine samples from COVID-19 patients will be analyzed, and biomarkers that could aid in identifying the infection and assessing the possible metabolic alterations caused by the SARS-CoV-2 virus in the body will be sought. The samples will be analyzed by GC-MS using three different preparations: untreated samples, lyophilized samples, and samples treated with H_2_SO_4_.

## 2. Materials and Methods

### 2.1. Study Participants

Patient recruitment and sampling procedures were conducted in accordance with the Declaration of Helsinki, as well as applicable local regulatory requirements and laws, and after receiving approval from the Ethics Committee of the Hospital Universitario de San Juan (Alicante, Spain). Written informed consent was obtained from each participant before inclusion in this study. Urine samples from COVID-19 patients were obtained from the Hospital de la Marina Baixa (Vila Joiosa, Alicante, Spain). The first set of COVID-19 patient samples (*n* = 20) was collected between September and November 2021, when the Delta variant was predominant in Spain. The second set of samples (*n* = 15) was collected between February and May 2022, when the Omicron variant was dominant. Healthy control samples were obtained from the University of Alicante personnel and outpatients from the Hospital de la Marina Baixa with another chronic and common illness (Vila Joiosa, Alicante, Spain) (Table 1).

### 2.2. Sample Preparation and Gas Chromatography–Mass Spectrometry (GC–MS) Analysis

Human urine samples (first pass, morning, with 10–14 h of fasting and at least 4 to 6 h since the last urination) were collected from volunteers in 120 mL sterile urine specimen cups. Upon receipt (typically within 1 h of collection), all samples were stored at −20 °C. Urine samples were stored for two weeks while all testing was performed. Prior to analysis, the samples were thawed at room temperature for 30 min and centrifuged at 12,000 rpm for 5 min. Afterward, samples were subjected to three different sample treatments, i.e., no treatment, lyophilization, and H_2_SO_4_ addition. Samples without treatment were analyzed directly [25]. The required amount of NaCl (0.270 g) was added to 1 mL of urine in a 10 mL glass vial with a micro-stirring bar (PTFE bar, 5mm diameter, 15mm length). For the lyophilized samples, 1 mL of urine was lyophilized before being placed in glass vials [26]. For the samples containing H_2_SO_4_, 0.2 mL of 2.5 M H_2_SO_4_ was added to 1 mL of urine in a 10 mL glass vial [25]. 

The glass vials were sealed with aluminum crimp caps equipped with a needle-pierceable polytetrafluoroethylene/silicone septa. The solid phase microextraction (SPME) fiber used was divinylbenzene/carboxen/polydimethylsiloxane (DVB/CAR/PDMS) 50/30 µm, StableFlex, 1 cm long, mounted on an SPME automatic holder assembly (Supelco, Bellefonte, PA, USA). The sample vial was then placed in a thermostatic water bath at 45 °C and stirred at 500 rpm for 30 min. After this equilibration, the SPME needle was exposed to the VOCs in the headspace for 30 min. Once the extraction was complete, the SPME needle was removed, and the fiber was immediately desorbed into the GC-MS injection port at 250 °C for 10 min. To avoid carryover, an additional step was added to our GC method. After analyzing each sample, the fiber was introduced into the oven with a helium flow (20 mL/min) for 2 min at 250 °C. Additionally, at the beginning and end of each sample processing batch, a blank analysis (empty, capped vials) was performed. 

Volatile analysis was carried out using an Agilent 6890N GC coupled to a 5973N MS (Agilent Technologies, Palo Alto, CA, USA) operating in electron impact ionization mode (EI 70 eV). The ion source and GC-MS transfer line temperatures were set to 230 °C and 280 °C, respectively. A DB-624 column (30 m length × 0.25 mm internal diameter × 1.4 µm film thickness, Agilent Technologies, Palo Alto, CA, USA) was used. The oven temperature was programmed as follows: 50 °C initial temperature with a hold for 3 min, then from 50 to 250 °C at 5 °C min^−1^, and finally hold for 10 min at 250 °C; the total run time was 53 min. Helium was used as the carrier gas (1 mL/min). Mass spectrum data were acquired in scan mode over a mass range of 30–350 mass-to-charge ratio (*m*/*z*), using MassHunter GCMS Acquisition B.07.06 (Agilent Technologies, Inc., 5301 Stevens Creek Boulevard. Santa Clara, CA, USA). Peak identification was based on a comparison of the mass spectrum data with spectra in the NIST/Wiley mass spectral library [27] using Enhanced ChemStation—MSD ChemStation F.01.03., Level 2 identification, according to the Metabolomics Standards Initiative (MSI) [28].

### 2.3. Statistical Analysis

GC-MS spectra were filtered for signals corresponding to the fiber and medications, such as the anesthetic Propofol, which was present in a great number of the samples from patients suffering from COVID-19 and hospitalized in intensive care units.

To better identify the VOCs, the raw data were exported to MATLAB R2024a [29] for mathematical processing. The maximum and minimum of the signals were marked, and an integration of these was performed, using, as a reference, the two minimum points on either side of peak’s maximum. Each segment was then reviewed to evaluate which signals were not artifacts due to baseline alterations in the chromatogram, nor were signals that appear only in urine samples from COVID-19 patients, which might indicate they are drugs used in treating the disease, such as propofol. We searched for signals present in both healthy individuals and COVID-19 patients that were sufficiently intense to enable correct classification.

Data analysis of the GC-MS spectra was performed using the PLS-LDA algorithm. PLS-LDA is a supervised method that groups data according to a mathematical model. This algorithm allows for the determination of whether data are correctly grouped and which properties (peaks in our GC-MS data) are important for accurate classification (COVID-19 patients or healthy controls). The statistical parameters used to determine the accuracy of the model were the R2Y, R2X, and AUC. Data were subjected to Pareto scaling prior to PLS-LDA; three components were selected [30,31]. The optimal number of latent variables of PLS-LDA was chosen by a five-fold cross validation [32]. 

In the GC-MS spectra, the peaks corresponding to VOCs were identified and integrated. The integrated data were classified with the SPA algorithm [32]. This algorithm ranks the variables based on the *p*-value. The PLS-LDA algorithm analyzes the ranking of each sample using a model created by excluding the sample in question from the complete set. This analysis is represented by tpScore [33]. We have confirmed that the model enables accurate classification across all samples and preparation methods for GC-MS.

## 3. Results

### 3.1. Samples Without Any Treatment

**Figure 1:**
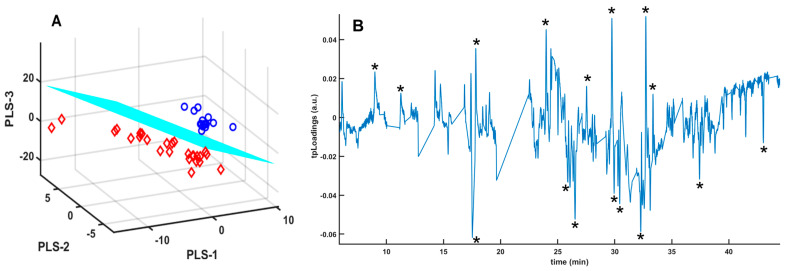
(**A**) Score plots of the three components of the PLS-LDA model for GC-MS spectra of urine samples from COVID-19 patients (red diamonds) and healthy controls (blue circles); (**B**) tpLoadings pseudospectrum from PLS-LDA model for GC-MS spectra of urine samples from COVID-19 patients and healthy controls. The intensity of the peaks (positive or negative) in the pseudospectrum is determined by the most significant spectral shift regions in the PLS-LDA model. The model performances were the following: R2X = 0.35, R2Y = 0.86, and AUC = 1. (*) Selected peaks appearing in Table 2.

First, we analyzed the urine samples directly [25], without any alteration except for adding a small amount of sodium chloride to adjust the ionic strength of the urine and promote the formation of VOCs. The PLS-LDA analysis was used to assess VOC features capable of classifying the urine samples in the group of COVID-19 patients or the group of healthy controls. The signals obtained were not of high intensity, and the spectra had to be processed to eliminate the signals generated by the fiber itself, which absorbs the VOCs during the analysis. However, they enabled us to achieve a good separation between the two groups analyzed, COVID-19 patients and healthy individuals (Figure 1). As shown on the score plots (Figure 1A), there is a good separation of the two groups. The pseudospectral representation of the tpLoadings indicates signals of the GC-MS spectrum which are most important in the separation observed in the PLS-LDA model (Figure 1B). The peaks of the negative tpLoadings correspond to compounds present at higher concentrations in COVID-19 patients, while the positive tpLoadings correspond to compounds found at higher concentrations in healthy individuals. These volatile compounds may appear or disappear in urine as a result of the metabolic alterations caused by COVID-19 [5,8].

GC-MS spectra allow for effective classification, but identifying which peaks correspond to the different VOCs was difficult. To improve identification, once the peaks were identified, we integrate the region where each peak is located. Before applying any mathematical classification model, we eliminate the integrals associated with peaks that appear due to the degradation of the fiber used to absorb volatile compounds in the GC-MS technique. The fiber is made of a silicon compound (polydimethylsiloxane), which is released during the analysis itself.

**Figure 2:**
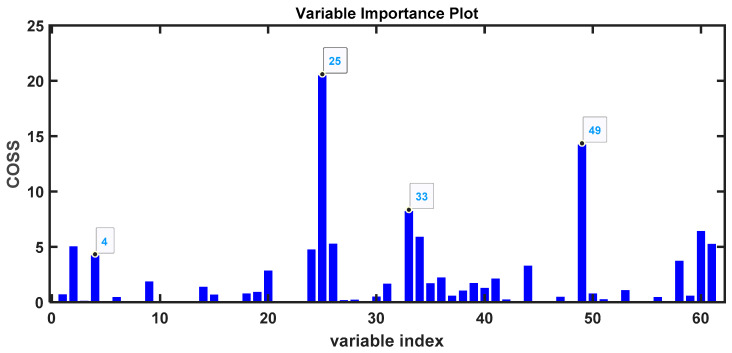
COSS values of the variables selected by the SPA algorithm as most important for determining the separation between groups of untreated urine samples from COVID-19 patients and healthy controls.

To identify the VOC peaks that differ between the groups of COVID-19 patients and healthy controls, we used the SPA (Subwindow Permutation Analysis) algorithm [32]. This algorithm identifies and classifies the values of the peak integrals that are most important for distinguishing between the groups. SPA is based on the COSS (Conditional Synergistic Score) value for variable selection (Figure 2). COSS is defined as -log10(*p*-value); thus, a *p*-value of 0.05 corresponds to a COSS value of 1.3. Several variables have much higher COSS values, indicating *p*-values much lower than 0.05 (Figure 2). 

**Figure 3:**
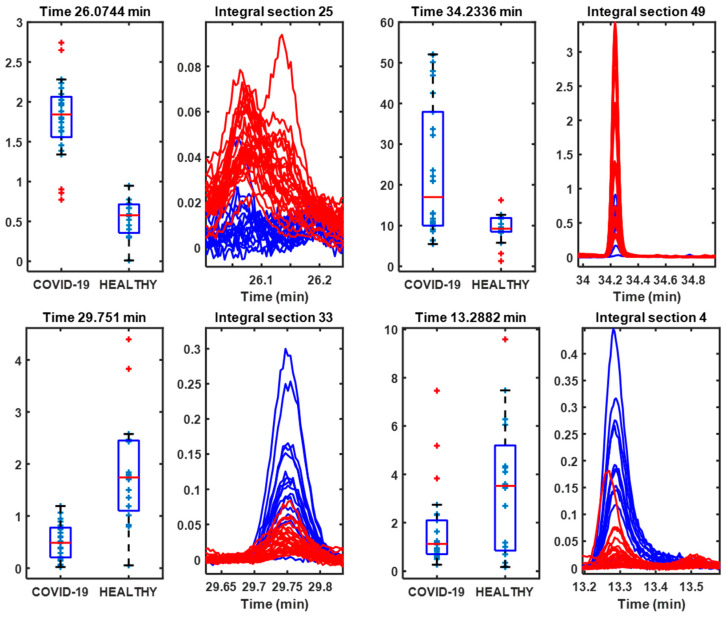
Boxplots of the integrals of the GC-MS spectrum regions and the peaks selected by the SPA algorithm as most important for determining the separation between COVID-19 patients and healthy controls.

Figure 3 shows the peaks contributing the most to the group separation, and their boxplot peak area. Among the peaks selected as most important following SPA classification, more intense peaks were found in both patients with COVID-19 and healthy controls, which would indicate alterations in metabolism [5,8]. 

Table 2 presents the 15 signals with the highest COSS values (lowest *p*-value), the compounds identified according to the Wiley library [27], the confidence percentage of the identification, and whether the compound was found at higher concentrations in COVID-19 or healthy controls group. These compounds are often present in urine samples and are associated with secondary metabolism or environmental contaminants [34,35,36,37,38]. In some samples of COVID-19 patients, signals associated with medications administered during hospital treatment were found, such as the anesthetic Propofol. Peaks associated with medication were eliminated from the statistical analysis.

**Table 2:** VOCs obtained from the analysis of untreated urine samples from COVID-19 patients and healthy controls. They are classified based on the SPA algorithm and their importance in classifying urine samples. The first four compounds in the table correspond to those shown in Figure 3.

Time (min)	*m*/*z*	Compound	Confidence (%)	Higher in
26.0744	164	2′-Hydroxy-4′,5′-dimethylacetophenona	87	COVID-19
34.2336	206	Phenol, 2,4-bis(1,1-dimethylethyl)	97	COVID-19
29.751	208	Decahydro-4,4,8,9,10-pentamethylnaphthalene	49	Control
13.2882	106	p-Xylene	97	Control
29.948	152	3-(But-3-enyl)-cyclohexanone	53	COVID-19
26.5193	135	Benzothiazole	94	COVID-19
9.5204	92	Toluene	95	Control
25.8843	72	1-(1-Propen-1-yl)-2-(2-thiopent-3-yl) disulfide	72	COVID-19
41.024	236	2,4-Diphenyl-4-methyl-2(E)-pentene	95	Control
24.4244	154	l-Menthone	98	Control
30.4316	252	5-Octadecene, (E)-	49	COVID-19
32.0588	170	Methacrylic acid, tetradecyl ester	74	COVID-19
17.4058	126	Dimethyl trisulfide	95	COVID-19
31.4391	200	1-Dodecanol, 2-methyl-, (S)-	80	COVID-19
30.2871	138	2-(2,2-Dimethylvinyl)thiophene	83	COVID-19

The variables selected by the SPA algorithm can be evaluated as potential biomarkers. Since the signal intensities were low, we set out to evaluate other strategies in order to increase both the signal generated by the volatile compounds and the number of volatile compounds generated. Sample lyophilization and sulfuric acid addition were further performed as two distinct sample treatments [25,26], and following the same data analysis procedure applied for samples without treatment.

### 3.2. Lyophilized Samples

**Figure 4:**
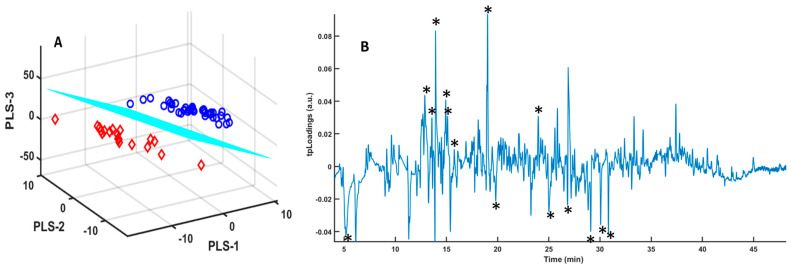
(**A**) Score plots of the three components of the PLS-LDA model for the GC-MS spectra of lyophilized urine samples from COVID-19 patients (red diamonds) and healthy controls (blue circles); (**B**) tpLoadings pseudospectrum from the PLS-LDA model for the GC-MS spectra of urine samples from COVID-19 patients and healthy controls. The intensity of the peaks (positive or negative) in the pseudospectrum is determined by the most significant spectral shift regions in the PLS-LDA model. The model performances were the following: R2X = 0.22, R2Y = 0.87, and AUC =1. (*) Selected peaks appearing in Table 3.

Urine samples were freeze-dried, as detailed in the Materials and Methods section. This method of sample pretreatment increases both the number of volatile compounds generated and the intensity of the signals, minimizing the impact of signals produced by the fiber on which the VOCs are adsorbed during the GC-MS analysis [26]. A good separation was obtained after PLS-LDA (Figure 4).

**Figure 5:**
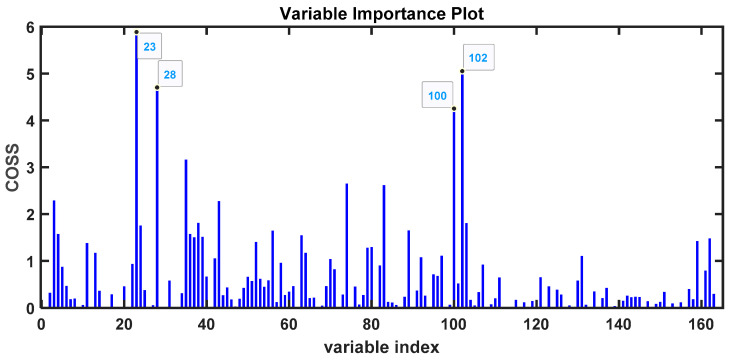
COSS values of the variables selected by the SPA algorithm as most important for determining the separation between groups of lyophilized urine samples from COVID-19 patients and healthy controls.

Although found to be less intense than in the analysis of untreated urine samples, signals corresponding to the fiber were also eliminated at this stage. We obtained a good selection of peaks, as illustrated in Figure 4B. Peak integrals were further obtained to improve the identification of urine samples from COVID-19 patients compared to those from healthy individuals. The data analysis included the SPA algorithm, which, with the COSS value, allows the selection of the most important variables (Figure 5) [32].

**Figure 6:**
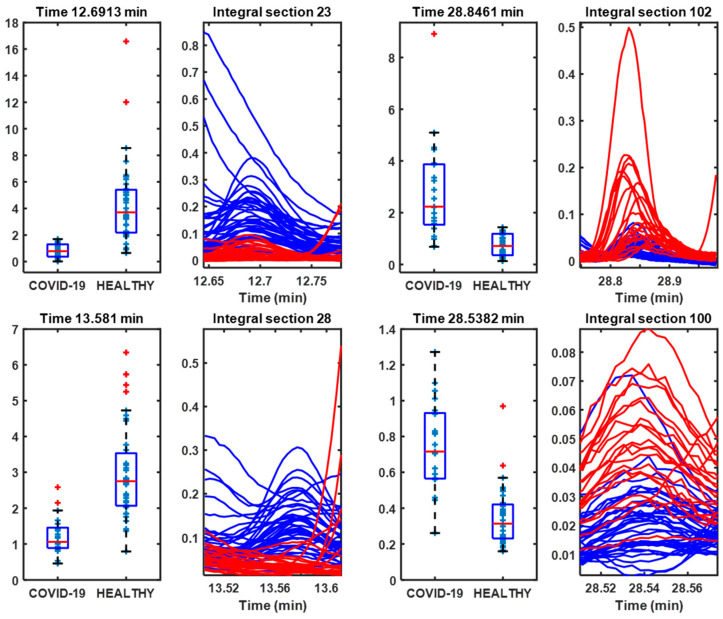
Boxplots of the integrals of the GC-MS spectrum regions and the peaks selected by the SPA algorithm as most important for determining the separation between COVID-19 patients and healthy controls.

The representation of the selected regions and the corresponding peaks allows for a better visualization of the different selected volatile compounds of interest with a boxplot peak area (Figure 6).

Table 3 presents the 15 signals with the highest COSS values for the lyophilized urine samples, the corresponding identified compounds according to the Wiley library [27], the confidence percentage of the identification, and whether the compound is found at a higher concentration in the COVID-19 group or the healthy control group.

**Table 3:** VOCs obtained from the analysis of lyophilized urine samples from COVID-19 patients and healthy controls. They are classified based on the SPA algorithm and their importance in classifying urine samples. The first four compounds in the table correspond to those shown in Figure 6.

Time (min)	*m*/*z*	Compound	Confidence (%)	Higher in
12.6913	90	2,3-Butanediol	80	Control
28.8461	113	Caprolactam	96	COVID-19
13.581	98	Cyclopentanone, 3-methyl-	96	Control
28.5382	148	Benzaldehyde, 2,4,5-trimethyl-	93	COVID-19
14.9345	78	Dimethyl Sulfoxide (DMSO)	96	Control
22.8465	42	2H-Pyran-2-one, tetrahydro-	90	Control
25.162	71	Oxalic acid, 2-ethylhexyl hexyl ester	64	COVID-19
5.1405	119	Methane-d, trichloro-	95	COVID-19
16.9914	170	Decane	93	Control
15.7596	96	2-Cyclopenten-1-one, 2-methyl-	94	Control
29.1009	158	Formamide, N,N-dibutyl-	97	COVID-19
12.851	90	2,3-Butanediol	90	Control
26.4242	135	Benzothiazole	93	COVID-19
19.8201	134	Benzene, 1,2,3,4-tetramethyl-	80	COVID-19
15.1208	108	Pyrazine, 2,6-dimethyl-	91	Control

### 3.3. H_2_SO_4_ Samples

**Figure 7:**
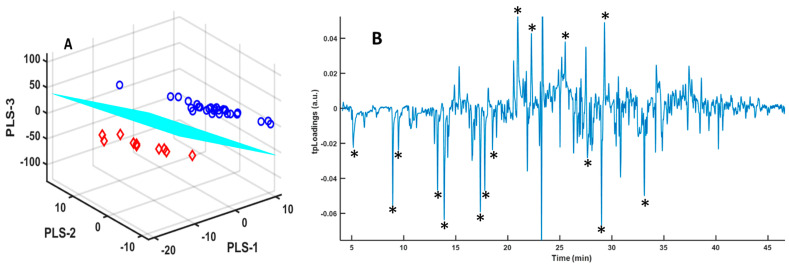
(**A**) Score plots of the three components of the PLS-LDA model for GC-MS spectra of H_2_SO_4_-treated urine samples from COVID-19 patients (red diamonds) and healthy controls (blue circles); (**B**) tpLoadings pseudospectrum from the PLS-LDA model for GC-MS spectra of urine samples from COVID-19 patients and healthy controls. The intensity of the peaks (positive or negative) in the pseudospectrum is determined by the most significant spectral shift regions in the PLS-LDA model. The model performances were the following: R2X = 0.25, R2Y = 0.77, and AUC =1. (*) Selected peaks appearing in Table 4.

The urine samples were treated with sulfuric acid, as detailed in the Materials and Methods section [25] with the aim of increasing both the number of volatile compounds generated and the intensity of the signals while minimizing the impact of the signals generated by the fiber itself, on which the volatile compounds are adsorbed during GC-MS analysis [25]. A good separation was obtained after PLS-LDA modelling (Figure 7).

As before, signals from the fiber were eliminated before building the model with PLS-LDA [33]. We obtained a good selection of peaks, as shown in Figure 7B, with a good R2Y value (0.77).

**Figure 8:**
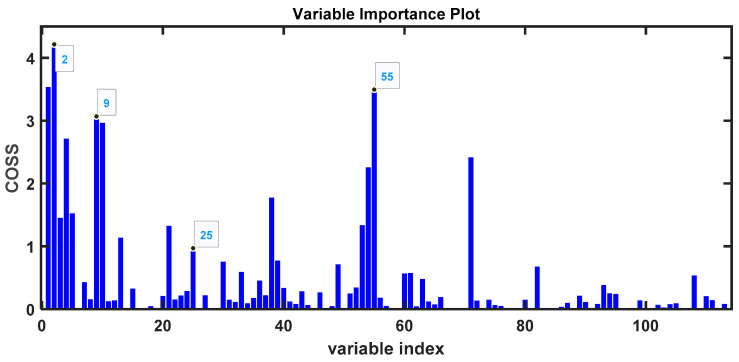
COSS values of the variables selected by the SPA algorithm as most important for determining the separation between groups of H_2_SO_4_-treated urine samples from COVID-19 patients and healthy controls.

To improve the selection and identification of variables associated with VOCs, we applied the SPA algorithm, which allows us to select the most important variables through the COSS value (Figure 8) [32].

**Figure 9:**
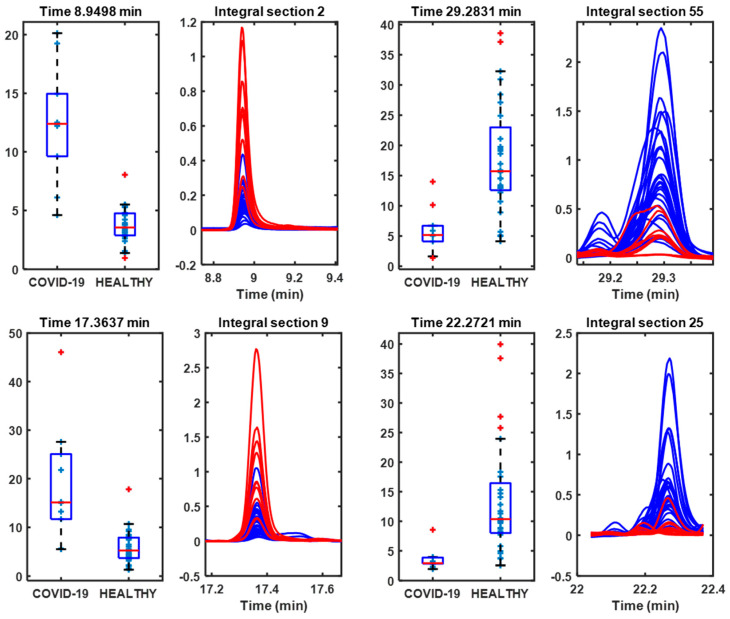
Boxplots of the integrals of the GC-MS spectrum regions and the peaks selected by the SPA algorithm as most important for determining the separation between COVID-19 patients and healthy controls.

This allows us to select a series of variables that correspond to the integral values (boxplot) of the peaks in the GC-MS spectrum of the urine samples (Figure 9).

Table 4 presents the 15 signals with the highest COSS values for urine samples treated with sulfuric acid, the corresponding identified compounds according to the Wiley library [27], the confidence percentage of the identification, and whether the compound is found at a higher concentration in the COVID-19 patient group or the healthy control group.

**Table 4:** VOCs obtained from the analysis of H_2_SO_4_-treated urine samples from COVID-19 patients and healthy controls. They are classified based on the SPA algorithm and their importance in classifying urine samples. The first four compounds in the table correspond to those shown in Figure 9.

Time (min)	*m*/*z*	Compound	Confidence (%)	Higher in
8.9498	94	Disulfide, dimethyl (DMDS)	97	COVID-19
29.2831	97	1,1,5-Trimethyl-1,2-dihydronaphthalene	97	Control
17.3637	126	Dimethyl trisulfide (DMTS)	97	COVID-19
22.2721	124	Phenol, 2-methoxy- (guaiacol)	94	Control
5.1212	84	Methane-d, trichloro-	94	COVID-19
17.7782	105	Benzene, 1,2,4-trimethyl-	90	COVID-19
13.8925	71	4-Heptanone	91	COVID-19
33.0889	204	Spiro[5.5]undeca-1,8-diene, 1,5,5,9-tetramethyl-, (R)-	98	COVID-19
29.1006	224	Cetene	97	COVID-19
25.4963	138	2-Methoxy-5-methylphenol	80	Control
14.44	74	Butanoic acid, 3-methyl-	81	COVID-19
9.4973	91	Toluene	95	COVID-19
29.0055	159	1H-Inden-1-one, 2,3-dihydro-3,4,7-trimethyl-	91	COVID-19
20.9414	170	trans-Linalool oxide (furanoid)	90	Control
18.5347	68	D-Limonene	98	COVID-19

## 4. Discussion

In 2010, it was proposed to use dogs for the detection of certain urological tumors and, during the 2020 pandemic, dogs were used to detect individuals infected with the COVID-19 virus. This suggested that the volatile compounds present in urine could be used as possible biomarkers of COVID-19 [19,39]. Changes in the composition of urine could be due to the metabolic alterations caused by SARS-CoV-2 infection [5,8]. Electronic noses could also be used to detect these volatile compounds in urine, but first, it is necessary to identify the biomarkers associated with the disease, as has been done in the detection of certain types of tumors [24,37,40]. Electronic noses also need to identify scent profiles to be effective, which would significantly limit their use in the case of COVID-19. In the present study, we have found a number of volatile compounds present in urine that could be considered biomarkers of COVID-19, using PLS-LDA models fit for discriminating individuals according to their status. The samples presented with an error rate of 0% and a sensitivity and specificity of 100% in all analyzed cases.

We performed three different treatments for the urine samples. Initially, a certain amount of NaCl was added to increase ionic strength and promote the formation of volatile compounds [25]. With the same objective, we freeze-dried the samples for the second treatment [26]. The third sample treatment consisted of sulfuric acid addition, as protonation of the molecules favors the formation of volatile compounds by decreasing the solubility of the protonated compounds [25]. In the first treatment, although the signal intensity was not very high, several compounds were identified (ID 2 according to the MSI), such as those listed in Table 2. The more intense signals in the urine of COVID-19 patients corresponded to 2′-hydroxy-4′,5′-dimethylacetophenone, 2,4-bis(1,1-dimethylethyl) phenol, and benzothiazole, which are aromatic compounds. In plants, compounds such as 2,4-bis(1,1-dimethylethyl) phenol appear in response to infections and are induced by arachidonic acid, playing an antioxidant role [41], but its role in human metabolism has not been identified. In healthy individuals, the signals corresponding to decahydro-4,4,8,9,10-pentamethylnaphthalene, toluene, and p-xylene were more intense. Some of these compounds, such as toluene, seem to have an exogenous origin, while others result from secondary metabolism [42,43,44], making it difficult to identify the metabolic pathways or precursors that generate them. We can assume that the differences between illness (COVID-19) and health generate distinct profiles in the spectra of volatile compounds, highlighting the metabolic alterations caused by COVID-19 [34,35,45,46].

For lyophilized samples, the spectra provided a greater number of volatile compounds and also allowed a good classification of the urine from COVID-19 patients using the PLS-LDA algorithm (Figure 4). When integrating the regions of the peaks, the SPA algorithm classifies these regions based on the *p*-value (Figure 5), allowing us to identify a series of potential biomarkers that are elevated in COVID-19 patients, such as caprolactam and benzaldehyde, 2,4,5-trimethyl, a biomarker in bladder cancer [47] or anxiety in mice [48]. In healthy individuals, we identified 2,3-butanediol; cyclopentanone, 3-methyl; and dimethyl sulfoxide (DMSO), or 2H-pyran-2-one, tetrahydro, as the most abundant compounds. 2,3-butanediol is a product of microbiota metabolism that contributes to reducing plasma cholesterol [49], and cyclopentanone has a protective effect against issues associated with diabetic nephropathy [50]. Other compounds, such as DMSO or 2H-pyran-2-one, tetrahydro, are products that do not form naturally and appear due to exposure [51]. 

With H_2_SO_4_-treated urine samples, another series of volatile compounds were found (Table 4). Some are present in higher proportions in samples from COVID-19 patients, such as disulfide, dimethyl (DMDS); dimethyl trisulfide (DMTS); benzene, 1,2,4-trimethyl-; and 4-heptanone. In healthy individuals, we predominantly have 1, 1, 5-trimethyl-1, 2-dihydronaphthalene; trans-linalool oxide (furanoid); or phenol, 2-methoxy- (guaiacol). As noted, many of these compounds may have an external origin, such as diet [34] or exposure to certain environments. For example, 1,2-dihydronaphthalene is related to exposure to naphthalene, although it can also be a product of the degradation of lutein or beta-carotene [42]. The fact that many volatile compounds have a dietary origin makes interpreting them as biomarkers for SARS-CoV-2 infection very challenging. Additionally, it is important to consider that the bacterial microflora also plays a significant role in generating these volatile compounds present in urine, such as DMDS [52,53,54]. The microbiota also appears to be altered during COVID-19. The enormous complexity of human metabolism, along with the microbiota [54], has been highlighted in studies analyzing volatile compounds in the urine of cancer patients [55,56]. 

As shown in Table 2, Table 3 and Table 4, the different pretreatments influence biomarker selection, favoring certain chemical families over others. In the analysis of untreated samples, hydrocarbons, ketones, organosulfur compounds, esters, and phenols are prominent. Without any additional treatment, this group presents a range of compounds that are inherently volatile and thermostable, making them suitable for GC-MS analysis. In the case of lyophilized samples, the primary compounds detected are alcohols, carboxylic acids, amines, lactams, and some halogenated compounds. The removal of water during lyophilization enhances the volatility of alcohols and carboxylic acids, improving their detectability in GC-MS [26]. Amines and nitrogen-containing compounds also benefit from lyophilization, as the process increases their concentration without altering their structure [26]. In contrast, samples treated with H_2_SO_4_ show the presence of phenols, organosulfur compounds, monoterpenes, ketones, and aromatic derivatives. The addition of sulfuric acid can protonate functional groups, particularly in phenols and amines, making some compounds more reactive [25]. This treatment can decompose unstable compounds, generating volatile derivatives or fragments that are detectable in GC-MS. Sulfuric acid may also induce dehydration in certain compounds, facilitating their volatilization [25].

Thus, while using a single sample treatment may be useful for creating a patient segregation model—allowing differentiation based on measurable changes in metabolites—a combination of different treatments is more valuable for a deeper study of disease-related metabolic alterations.

## 5. Conclusions

The analysis of urine samples using HS-SPME-GC–MS reveals differences between samples from COVID-19 patients and healthy individuals. These differences are clearer when methods enhancing the formation of volatile compounds, such as sample lyophilization or sulfuric acid addition, are used. 

We have identified a number of compounds whose concentration could serve as biomarkers, but further studies for biomarker confirmation using reference standards are required, as well as biomarker confirmation in an independent cohort, in order to ascertain their use in COVID-19 infections.

It is also important to note that a single sample preparation method for GC-MS may be sufficient to classify patients as healthy or diseased but is very limited for studying metabolic alterations. Therefore, using a combination of methods may be more informative when evaluating metabolic alterations caused by viral infections and would allow for a better selection of biomarkers. 

## Figures and Tables

**Table 1:** Population and clinical data of the people whose urine have been analyzed.

	Healthy Controls (*n* = 32)	COVID-19 Patients (*n* = 35)
**Age [Median (IQR)]**	52.5 (19.1)	59 (20.0)
**Sex, distribution**		
Male [*n* (%)]	9 (28.1)	11 (31.4)
Female [*n* (%)]	23 (71.9)	24 (68.6)

## Data Availability

The data presented in this study are openly available in Marhuenda, Frutos (2024), “Urine GC-MS COVID-19”, Mendeley Data, V2, https://doi.org/10.17632/j3szbwjnnd.2.
